# The amyloid fold of Gad m 1 epitopes governs IgE binding

**DOI:** 10.1038/srep32801

**Published:** 2016-09-06

**Authors:** Rosa Sánchez, Javier Martínez, Ana Castro, María Pedrosa, Santiago Quirce, Rosa Rodríguez-Pérez, María Gasset

**Affiliations:** 1 Instituto de Química Física “Rocasolano”, Consejo Superior de Investigaciones Científicas, 28006 Madrid, Spain; 2 Instituto de Química Médica, Consejo Superior de Investigaciones Científicas, 28006 Madrid, Spain; 3 Departamento de Alergología, Hospital Universitario La Paz, 28046 Madrid, Spain; 4 Instituto de Investigación-Hospital Universitario La Paz (IdiPaz), 28046 Madrid, Spain

## Abstract

Amyloids are polymeric structural states formed from locally or totally unfolded protein chains that permit surface reorganizations, stability enhancements and interaction properties that are absent in the precursor monomers. β-Parvalbumin, the major allergen in fish allergy, forms amyloids that are recognized by IgE in the patient sera, suggesting a yet unknown pathological role for these assemblies. We used Gad m 1 as the fish β-parvalbumin model and a combination of approaches, including peptide arrays, recombinant wt and mutant chains, biophysical characterizations, protease digestions, mass spectrometry, dot-blot and ELISA assays to gain insights into the role of amyloids in the IgE interaction. We found that Gad m 1 immunoreactive regions behave as sequence-dependent conformational epitopes that provide a 1000-fold increase in affinity and the structural repetitiveness required for optimal IgE binding and cross-linking upon folding into amyloids. These findings support the amyloid state as a key entity in type I food allergy.

Amyloids are polymeric states of proteins defined by a cross-β sheet backbone, in which β-sheets that are mostly formed from β-strands of different molecules pack through their side chains^1^,^2^,^3^,^4^. Despite their initial association with proteins involved in neurodegenerative disorders, an increasing number of proteins that form functional and pathogenic amyloids have been reported^1^. Indeed, protein chains containing sequences with cross-β sheet folding traits under conditions that cause their solvent exposure enter this state when they are present at sufficient concentrations to overcome the entropy that opposes fibril ordering. Amyloids exhibit a dual (soluble/insoluble) nature and a characteristic structural repetitiveness, allowing phase separations and interactions with enhanced affinity and specificity compared to the precursor monomer^2^,^3^,^5^,^6^,^7^. These latter properties argue that allergen fibrillization could sculpt non-native epitopes and dictate the valence and affinity parameters of the IgE interaction in type I food allergies, which are key features of effector cell activation.

Fish β-parvalbumins represent the major allergens of IgE-mediated fish hypersensitive patients^8^,^9^,^10^,^11^. β-Parvalbumins are 12 kDa calcium-binding proteins with three EF hand motifs; a nonfunctional AB motif, followed by the CD and EF Ca^2+^-binding motifs^12^,^13^,^14^,^15^,^16^. Despite the variations in sequence identity between proteins from the same and different fish species, β-parvalbumins display a high IgE cross-reactivity, supporting a pattern of recognition other than the sequence identity^9^,^12^,^17^,^18^,^19^,^20^. The use of a variety of approaches, including protease digests, phage display libraries, site-directed mutagenesis and arrays of overlapping peptides with β-parvalbumins from *Baltic cod* (Gad c 1)^21^,^22^,^23^,^24^,^25^, *Atlantic cod* (Gad m 1)^26^, *Common carp* (Cyp c 1)^10^,^27^,^28^,^29^,^30^, *Chub mackerel* (Sco j 1)^31^ and Atlantic salmon (Sal s 1)^32^,^33^, has shown distinct IgE epitopes of both linear and conformational types. For Gad c 1 and Sal s 1, the IgE binding sites have been located at the spacers between the AB and CD motifs (residues 28–45) and CD and EF motifs (residues 65–74), whereas for Gad m1, the dominant IgE binding region was found at the C-terminus (segment 95–109)^26^,^32^. On the other hand, disruption of the Ca^2+^ binding sites by mutagenesis in both Cyp c 1 and Sco j 1 resulted in soluble chains with reduced IgE-binding signals^30^,^34^,^35^. Conversely, stabilization of apo-Gad m 1 produces highly stable amyloids with enhanced IgE reactivity^36^.

Here, we have addressed the role of Gad m 1 amyloid with respect to both its IgE-reactive properties and epitope architecture. Using surface-bound peptide arrays of Gad m 1, sera from fish allergic patients, recombinant wt and mutant Gad m 1 (rGad m 1) chains and biophysical approaches, we found that the relevant antigenic regions of the Gad m 1 chain fold into amyloids and, by doing so, optimize the IgE interaction.

## Results

We first characterized the sequence elements to address how amyloid formation contributes to the IgE reactivity of Gad m 1. A set of 12-mer overlapping synthetic peptides with an offset of 2 that correspond to the sequence of Gad m 1 was used in an array-based immunoassay (Supplementary Fig. S1). This peptide length and surface density (10 nmol/spot) ensures the preservation of amyloid formation (which is conventionally limited to 6 residues), and differs from previous microarrays assays using a 15-mer with an offset of 3^26^.

**Figure 1:**
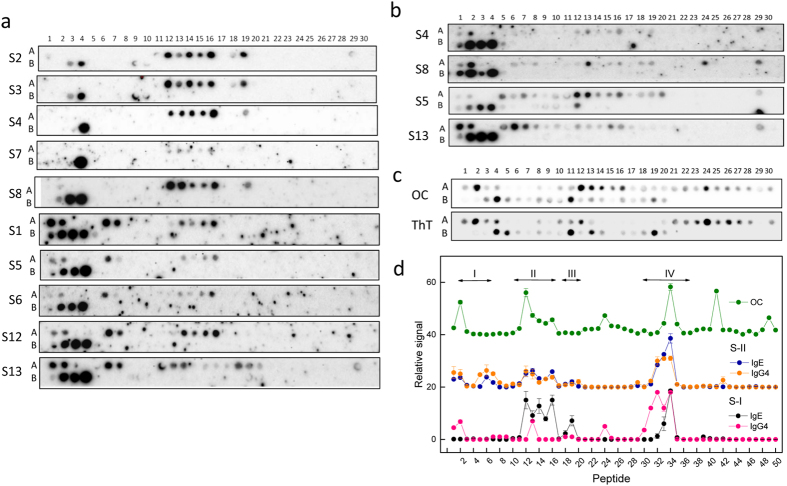
Reactivity of Gad m 1 chain. (**a**) Regions binding IgE in sera from fish-allergic patients. (**b**) Regions binding IgG4 in sera from fish-allergic patients. (**c**) Sequences with anti-amyloid OC antibody reactivity and ThT binding properties. (**d**) Relative signal of the binding of IgE and IgG4 from the sera of fish-allergic patients and the anti-amyloid OC antibody to the distinct overlapping peptides representing the Gad m 1 sequence. Signals corresponding to the IgE and IgG4 binding are displayed as the average and standard deviations of the signals of sera groups S-I (S2, S3, S4, S7, and S8) and S-II (S1, S5, S6, S12, and S13) are shown. Peptides are indicated by numbers on the top (row A: peptides 1–30, row B: peptides 31–50) and their sequences are displayed in Supplementary Fig. S1. The sera are depicted as Si, where i is a number (Supplementary Table S1).

**Figure 2:**
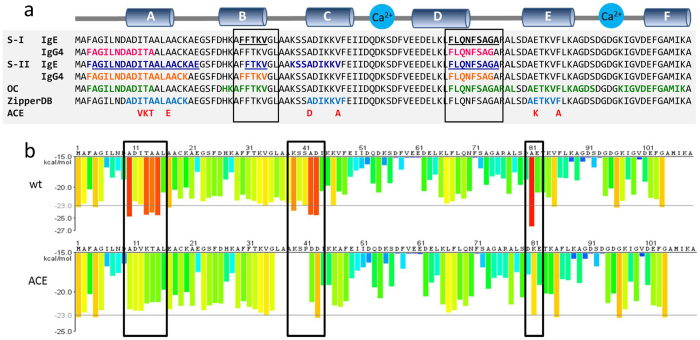
Location and relation of the immunoreactive and amyloid forming regions in the Gad m 1 chain (**a**) Sequence and location of the following regions in the Gad m 1 chain: ?(i) IgE-binding epitopes found in sera groups S-I (black and underlined) and S-II (navy blue and underlined), (ii) IgG4-binding epitopes found in S-I (pink) and S-II (orange), (iii) OC-binding segments (green), (iv) adhesive regions identified by the ZipperDB algorithm (light blue), and (v) sequence changes in the A, C and E regions (red). The sequence changes were based on the sequences of the following β-parvalbumins: (i) Q91482, Q90YK8 and E0WDA2 for A, (ii) Q90YL0, Q91482, Q91483, E0WDA2 and Q90YK8 for C, and (iii) C6GKU7 for E^36^. **(b)** Comparative ZipperDB analysis of the Gad m1 wt and ACE mutant chains. Red bars indicate the N-terminus of a hexapeptide with adhesive properties. The effects of the adhesive segments are depicted by rectangles and were conserved in the analysis of chains containing single A, C and E modifications.

The peptide membranes were assayed using the sera from ten patients who are allergic to fish (Figs 1 and 2). The IgE binding intensity was variable among the tested sera; however, it was possible to identify four major binding regions (I–IV) and two major groups of sera (S-I: S2, S3, S4, S7 and S8; S-II: S1, S5, S6, S12 and S13). Region I was only recognized by the sera group S-II and covers peptides 1–2 (FAGILNDAD common core) and 6-7 (TAALAACKAE common core). Region II, which was constituted of peptides 12–16, with FTKV as the common core, was identified by all sera with a high relative signal. In contrast, region III formed by peptides 18-19 (AAKSSADIKK common core) displayed variable recognition by the distinct sera. Region IV covering the peptides 31-34 (FLQNFS common core) displayed high intensity, but varied between the two sera groups. Of these four regions, region I overlaps the sequence predicted as the cross-β sheet forming (adhesive) segment in helix A, whereas regions III and IV overlap the immunologically reactive sites located on the junctions between the AB and CD domains (residues 33–44) and between the CD and EF domains (residues 65–74)^29^,^36^. Nevertheless, the reactivity of the Ca^2+^-binding loop of the EF domain (residues 88–96) and of the C-terminal (residues 95–109) regions was not detected by these sera, as probed by both IgE and IgG_4_ binding (Fig. 1d)^26^,^32^. It merits comment that IgG_4_ reactivity, which is considered as a protective response in allergy, is largely detected in region IV for both sera groups^26^,^32^.

The presence of amyloid folds was then assayed using the conformation-selective OC antibody, which detects oligomer and fibril assemblies produced during the protein fibrillization process and the amyloid probe thioflavin T (ThT)^37^,^38^,^39^. Figure 1c shows that OC immunoreactivity covers peptides 2 (FAGILNDADITA), 12–16 (preferentially sequences containing FTKV as the common core), 24 (VFEIIDQDKSDF), 34 (FLQNFSAGARAL), 41 (AETKVFLKAGDS) and 49 (KIGVDEFGAMIK). Of these regions, peptides 2 and 41 overlap the adhesive segments in helix A and E, predicted by the ZipperDB algorithm^1^,^36^, peptides 12–16 and 34 lie over the IgE-binding regions III and IV, peptide 24 contains part of the Ca^2+^-binding site of domain CD, and peptide 49 corresponds to previously described epitope groups^26^,^32^ (Fig. 2 and Fig. S2). Similar to the OC reactivity, probing the membranes with ThT shows increased fluorescence emission for peptides 2, 12, 24, 34 and 41, confirming the presence of amyloid folds^3^. It must be noted that peptides containing the helix C sequence, which was predicted to be an adhesive segment, were not detected by either the OC or ThT binding assays.

**Figure 3:**
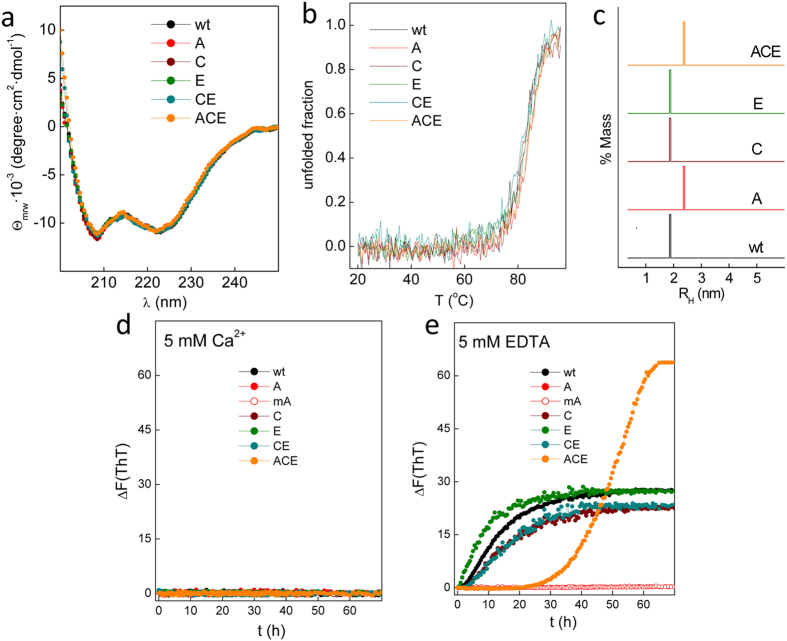
Conformational features of rGad m 1 wt and its ACE mutants. **(a)** Far-V Circular Dichroism spectra of the distinct proteins obtained in 50 mM Tris-HCl, pH 7.5, containing 2 mM Ca^2+^ at 25 μM protein concentrations. (**b**) Variation of the unfolded fraction of rGad m 1 wt and mutants with temperature. **(c)** DLS determination of the hydrodynamic radius (R_H_) of rGad m 1 wt and the mutants. The measurements were performed in 50 mM Tris-HCl, pH 7.5, containing 2 mM Ca^2+^ at 160 μM protein concentrations. Amyloid formation was monitored by the time-dependence of ThT binding of rGad m 1 wt in 50 mM Tris-HCl, pH 7.5, in the presence of **(d)** 5 mM Ca^2+^ or **(e)** 5 mM EDTA.

Taken together, these results indicate that the Gad m 1 chain contains two major IgE binding sites (regions II and IV), which are flanked and overlapped by sequences with amyloid-forming capacity (Fig. 2a). We modified the sequences with a predicted amyloid-forming capacity identified by the ZipperDB analysis as regions A, C and E to uncouple amyloid formation from IgE binding without disrupting the linear epitopes (Figs 2a and 3). To preserve the native fold we searched for different sequences in the A, C and E regions of β-parvalbumins from other fish species with commercial added value and tested the effect of substitutions by a second round of ZipperDB analysis^4^,^36^. Figure 2b shows that the I12V-T13K-A14T-A17E mutations in region A, A42D-V47A mutations in region C, and E82K-F86A mutations in region E abrogate the predicted adhesive properties of each of the segments of the wt chain. All of the chains containing the single or combined mutations in regions A, C and E were produced as recombinant proteins, and their conformations were characterized. All chains yielded Ca^2+^-bound folds with predominant α-helical secondary structures and a highly stable cooperative fold with a denaturation temperature of approximately 85 °C (Fig. 3a,b). These data indicate that the considered single (A, C, and E mutants) and combined (ACE mutant) mutations in regions A, C and E preserve the conformation and stability of the wt protein, in agreement with their chimeric-like trend. However, the DLS analysis of their hydrodynamic features shows that the wt C and E chains yielded an R_H_ value of 1.9 ± 0.02 nm, similar to the R_H_^T^ of a spherical monomer, whereas mutants A and ACE displayed an R_H_ value of 2.4 ± 0.04 nm, indicating a less compact fold (Fig. 3c).

Based on this conformational pattern, the capacity of the different chains to form amyloid aggregates was analyzed using the ThT binding assay (Fig. 3d,e). As previously shown, incubating 150 μM rGad m 1 at pH 7.5 in the presence of EDTA produces an increase in ThT fluorescence as a result of fibrillization, whereas in neutral media and in the presence of Ca^2+^, the fluorescence of ThT remains unaltered^36^. Similar to rGad m 1 wt, the ThT fluorescence readings of all mutant chains at pH 7.5 in the presence of Ca^2+^ remained unaltered for at least 70 h (Fig. 3d). In contrast, in the presence of EDTA, the distinct rGad m 1 mutants showed altered fibrillization: mutant E showed slightly faster fibrillization, mutants C and CE showed reduced fibrillization, and fibrillization of mutant A was abolished (Fig. 3e). The impaired amyloid formation observed in the A mutant was preserved upon cleavage of the N-terminal histidine tail, excluding tag effects, as shown for the wt chain^36^. Interestingly, the ACE mutant displays a largely retarded (lag phase of approximately 24 h) but highly cooperative fibrillization at approximately 60 h (Fig. 3e). Therefore, the adhesive region A largely governs rGad m 1 amyloid formation and its modification provides a non-amyloid-forming chain (A mutant) and a highly retarded amyloid-forming form (ACE mutant) as function of the sequences in the regions C and E.

**Figure 4:**
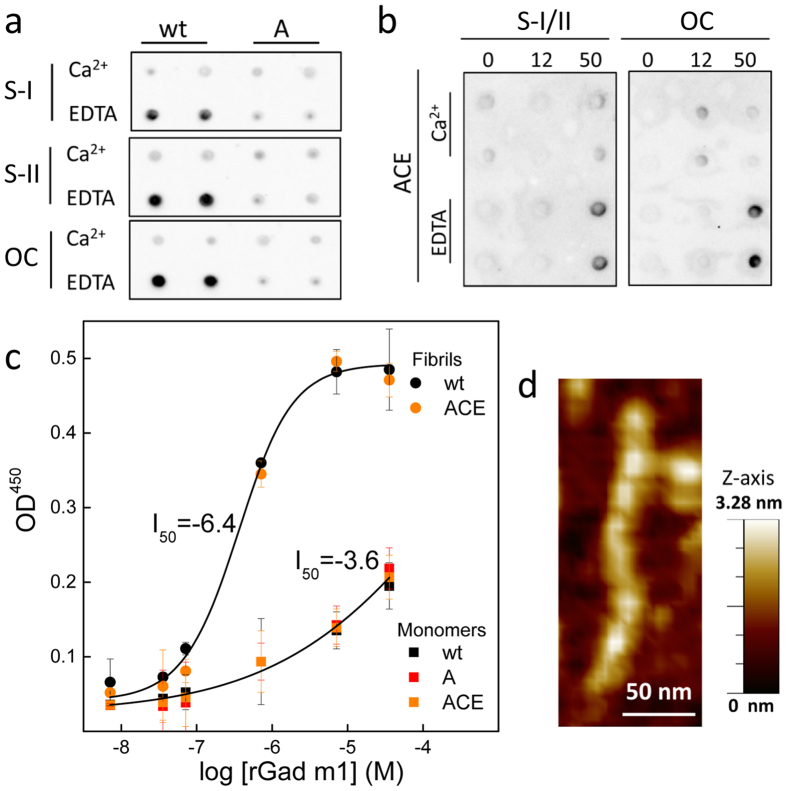
rGad m 1 amyloids displayed enhanced interaction with the IgE in the sera from fish-allergic patients. **(a)** Dot-blots of rGad m wt and its non-amyloidogenic A mutant probed with the sera pools S-I and S-II and with the anti-amyloid OC antibody. Typically, 50 ng of proteins that were incubated for 50 h under monomer-preserving (5 mM Ca^2+^) and amyloid-forming (5 mM EDTA) conditions were spotted. (**b**) Dot-blots of the ACE mutant at 0, 12 and 50 h of incubation in 50 mM Tris-HCl, pH 7.5, containing 0.1 M NaCl and either 5 mM Ca^2+^ or 5 mM EDTA were probed with a sera pool (S-I/II) and the anti-amyloid OC antibody. **(c)** Titration curves of rGad m 1 wt and of its A and ACE mutants stabilized in the monomer (M) and amyloid (F) states with sera IgE generated by ELISA assays and their best fits to sigmoidal curves. The data are displayed as the mean average value of two experiments performed in duplicate and their standard deviations. **(d)** AFM image of a typical amyloid assembly of rGad m 1 and its z-axis dimension.

We first used dot-blot assays to analyze the binding features of the different states to test the effect of amyloid formation on the IgE interaction (Fig. 4a,b). Amyloids obtained after 70 h of incubation of rGad m 1 wt in 5 mM EDTA exhibited an at least 50-fold enhancement of IgE binding compared to the Ca^2+^-bound monomer. This enhancement was not observed with the amyloid-prone A mutant under similar conditions, and it was rescued in the ACE mutant at long incubations under the assembly conditions (Fig. 4a,b). Therefore, the dot-blot assays support the hypothesis that amyloid assembly endows rGad m 1 with enhanced IgE-binding activity. We developed an ELISA assay to quantify the observed differences in binding. In this assay, the monomers and amyloid states of the distinct rGad m 1 chains were immobilized at varying concentrations and their IgE binding was determined using a sera pool (Fig. 4c). Under the conditions used, both the monomers and amyloids displayed similar adsorption levels, as shown by the protein concentration determinations, allowing the assignment of signal differences to the binding process. Amyloids prepared from rGad m 1 wt and the ACE mutant yielded I_50_ values of approximately 10^−6.4^ M, whereas titration of the distinct monomers (wt, A and ACE in 5 mM Ca^2+^) resulted in I_50_ values of approximately 10^−3.6^ M. Using I_50_ as the apparent dissociation constant (K_Dapp_), these values then yield K_Dapp_ of 4 × 10^−7^ M and 2 × 10^−4^ M for the amyloid and monomer forms, respectively. These data reveal that amyloid assembly provokes a 1000-fold enhancement of IgE affinity. On the other hand, using the dimensions of the rGad m 1 fibrils from the AFM images (2 nm height, 15 nm diameter and an average length of 350 nm) (Fig. 4d), the R_H_ value of the monomer from DLS measurements (Fig. 3c) and by applying simple geometrical considerations, it can be calculated that the average number of monomers per aggregate is 370. Using this aggregation number, the amyloid K_Dapp_ amounts to 1 × 10^−9^ M, supporting a tight interaction.

**Figure 5:**
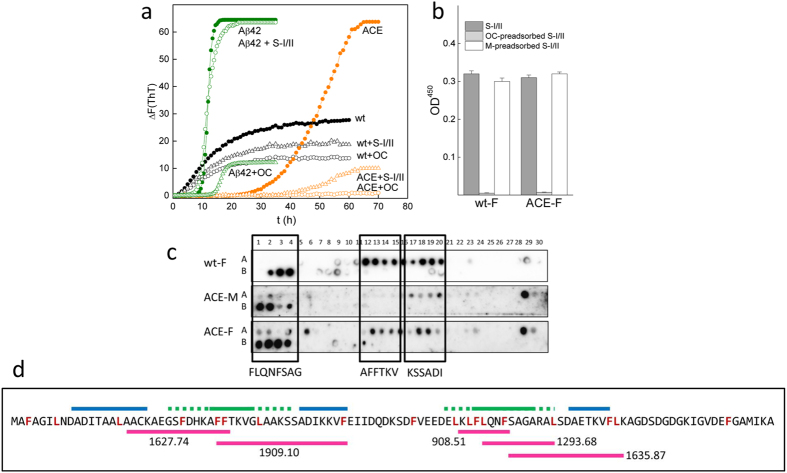
Gad m 1 sequences that are recognized by IgE in patient sera display amyloid folds. **(a)** The effects of patient sera (S-I/II) and the anti-amyloid OC antibody on the amyloid formation kinetics of rGad wt (black), its ACE mutant (orange) and Aβ42 (green) were monitored by ThT-binding in 50 mM Tris-HCl, pH 7.5, containing 0.1 M NaCl and 5 mM EDTA. **(b)** Effects of the anti-amyloid OC antibody and Gad m 1 monomers on recognition of the wt and ACE amyloid fibrils by the IgE in patient sera, as determined by the ELISA assay. Amyloid fibrils (100 ng/well) were probed with the patient sera pool that had been pre-adsorbed with 1 μg of BSA (S-I/II), 1 μg of OC (OC-preadsorbed S-I/II) and 1 μg of the corresponding monomer chain (M-preadsorbed S-I/II). **(c)** Gad m1 peptide arrays were probed with amyloids (wt-F and ACE-F) and monomers (ACE-M) and developed with the anti-His tag antibody to detect binding. **(d)** Sequence coverage of rGad m 1 wt obtained by LC-MS/MS analysis of the peptide mixture after pepsin digestion of the amyloids. The pepsin cleavage sites are displayed in red, the predicted adhesive regions are shown as blue lines, and the IgE binding regions are indicated with green lines (solid in the common core, dotted for all peptides). The sequences of the overlapping IgE-binding regions in the peptides are depicted in pink and their mass/charge ratio (m/z) is indicated nearby.

The differences observed in the K_Dapp_ of the sera IgE binding to monomers and amyloids cannot simply be explained in terms of the polymer structural repetitiveness. We assayed the effect of sera on amyloid formation to analyze whether amyloid formation sculpts the architecture of nonnative epitopes, accounting for the high IgE affinity. Figure 5a shows that sera, like the anti-amyloid OC antibody, disrupt amyloid formation in both rGad m 1 wt and ACE, as judged from the decrease in the final ThT fluorescence intensity. Sera inhibition is specific for Gad m 1 chains because the fibrillization of Aβ42 control is only diminished by the anti-amyloid OC but remains unaffected by the presence of sera. Similarly, pre-incubation of sera with OC, but not with monomers, abrogates the binding of IgE to rGad m 1 wt and ACE amyloids (Fig. 5b). Therefore, both results indicate that sera IgE and OC compete for the recognition of Gad m 1 amyloids, which agrees with the proximity and overlap of the epitopes (Fig. 2a). We exploited the properties of the amyloid formation mechanism and searched for Gad m 1 sequences involved in fibril growth to separate the contributions from proximity and overlap and rule out steric effects^40^. For this purpose, amyloid fibrils formed by wt and ACE chains and the ACE monomers were incubated with the peptide arrays and binding was determined using the anti-6XHis antibody, which recognizes the tag present in the recombinant chains. Figure 5c shows that the amyloid fibrils differentially bound to peptides 12–15 compared to the monomers, indicating that the GSFDHKAFFTKVGLAAKS sequence is a reactive segment for amyloid growth. Because this segment matches the IgE-binding region II (Fig. 1), these results support the sequence overlap of the antigenic and amyloid-folding activities.

We took advantage of the protease resistance of amyloids to isolate and identify the assembly core and strengthen the observed functional overlap^36^. Given the presence of several pepsin cleavage sites at the IgE-binding regions and the stability of the assemblies at pH 1.3^36^, the rGad m 1 wt amyloids were extensively digested with pepsin and the resulting fragments were analyzed by mass spectrometry (Supplementary Figs S2 and S3). Figure 5d shows that among the peptides detected, the sequences AACKAEGSFDHKAFF, FTKVGLAAKSSADIKKVF, KLFLQNF, FLNQNFSAGARAL and SAGARALSDAETKVFL contain protected pepsin-cleavage sites and match the IgE-binding regions II and IV. Therefore, these data support the hypothesis that the sequential IgE epitopes of Gad m 1 encrypt an amyloid fold, indicating their functions as sequence-dependent conformational epitopes.

## Discussion

Despite the large number of sequences and 3D structures of allergens elucidated in the last few decades, the knowledge of how allergens recognize and cross-link cell-bound IgEs is still limited^41^,^42^. This lack of knowledge is largely due to the complexity of allergen epitopes and their incomplete interpretation, based on the use of structures obtained from stable and ligand-bound states^26^,^32^,^43^,^44^. For food allergens that undergo drastic environmental changes during gastrointestinal transit, these structural templates are merely snapshots of the protein life and other nonnative structures can be formed and change their interaction repertoire. One such structural state is the amyloid state that confers a polymeric trait required for multivalent interactions through the spine structure and growth properties. Amyloid formation is a sequence-dependent process and has been described for distinct food allergens, such as Bos d 5 (β-lactoglobulin), Bos d 10 (κ-casein), Bos d 12 (αs2-casein), Gad m 1 (β-parvalbumin), Gal d 2 (ovalbumin), and Gal d 4 (lysozyme), among others^36^,^45^,^46^,^47^,^48^,^49^,^50^. Of these processes, only Gad m 1 amyloidogenesis was studied from the allergenic point of view, revealing the IgE binding capacity of the assemblies^36^,^45^,^46^,^47^,^48^,^49^,^50^. Here, we have found that the amyloid state of Gad m 1 is essential for its ability to bind IgE; the results provided both the architecture of the epitopes and the repetitiveness required to optimize the interaction parameters, such as affinity and multivalence. This finding identifies allergens with a previously unconsidered sequence-dependent reactive conformation and adds a novel discrete function for the amyloid state.

The search for regions coding Gad m 1 amyloid assembly showed a complex chain organization. On the one hand, the ZipperDB algorithm putatively identified three regions (A, C and E) with favorable energetic fits for steric zipper formation. However, of these regions, only A and E exhibit OC-antibody binding as peptides. According to the ThT binding kinetics, region A plays a role in the initial steps of the polymerization process (namely, the nucleation step) with an outcome that is dependent on the sequences of the C and E regions. On the other hand, the regions identified as the two major IgE linear epitopes that formed part of pepsin-resistant fragments and displayed OC binding properties escaped the ZipperDB prediction analysis. The failure of the ZipperDB prediction suggests that these segments form cross-β spines that are different than the model steric zipper used as a reference in the algorithm. In fact, spines consisting of parallel β-turns resulting from β-hairpins that are distinct from the steric zipper model have been described for other amyloids^51^. In addition, the interdependence of the assembly process on the A, C and E sequences suggests a role for long-range effects and the possibility of cross-β spines formed by distinct β-sheets, which are not considered in the algorithm^1^,^2^,^3^.

Gad m 1 amyloids are formed from the apo form and their IgE reactivity argues against the well-established Ca^2+^-binding dependence of β-parvalbumins-IgE interaction^30^,^36^,^52^. However, this contradiction is apparent and both facts converge when the IgE interaction is analyzed in terms of the physical state of the allergen. First, the results obtained here using the wt, A and ACE chains of Gad m 1 show that the structural impact of Ca^2+^ removal is highly dependent on the chain sequence, as amyloid formation was triggered in wt and the ACE mutant, but not in the A mutant. Therefore, these data indicate that not all apo forms will form amyloids. Second, the hypoallergenic mrCyp c 1 with the two mutated Ca^2+^ binding sites exhibits a highly stable native fold that argues against its capacity to polymerize^52^. Therefore, β-parvalbumin chains that are unable to form amyloids share a diminished IgE interaction. Third, the overlap of epitopes and amyloid folds in the AB motif, which is a hot spot for conformational exchange and lacks Ca^2+^-binding properties, suggests that amyloids could also form in the presence of Ca^2+^ as a function of the chain sequence^53^,^54^. Therefore, unless specifically removed by centrifugation, β-parvalbumin solutions may contain traces of amyloid species that, as function of their analytical use, could mislead reactivity assignments.

The finding of amyloid folds for Gad m 1 IgE epitopes agrees with the unusual structural features of the Bos d 5/Fab immunocomplex^44^. In this complex, the epitope of the milk allergen Bos d 5 was observed as a flat β-sheet resembling a monomer unit of a cross-β sheet^44^. Nevertheless, the determination of the structure of the allergenic cross-β sheet motif will require the use of specific approaches, such as X-ray crystallography of short fragments and solid state NMR, among others^4^,^55^,^56^. Such structures will provide the identities of the β-strand segments, the number and organization of the strands in the β-sheets, and the conformations of the non-β-strand segments. This information will be essential for the identification of as yet unconsidered targets for a potential pharmacological intervention.

Both Gad m 1 monomers and amyloids bind IgE, but with significantly different affinities. The binding of sera IgE to monomers features a high K_Dapp_ (mM range). The weakness of this interaction agrees with the loose contacts observed in the Bos d 5/Fab immunocomplex structure^44^. In contrast, the interaction of sera IgE with Gad m 1 amyloids is featured by a K_Dapp_ in the μM range, revealing a 1000-fold enhancement of the interaction strength compared to the monomer. This tightening of the interaction can be explained by several factors. From a structural point of view, the folding of the epitope into a cross-β sheet may optimize the number and nature of contacts for optimal antibody recognition. From the stoichiometry point of view, the polymer nature of amyloids permits the saturation of the two IgE binding arms, whereas a monomer does not permit saturation. Moreover, binding of the first IgE arm consists of an intermolecular reaction, whereas the occupancy of the second site involves an intramolecular reaction.

On the other hand, allergens require the existence of at least two IgE epitopes for their deleterious IgE cross-linking on the surface of effector cells. This requirement presumes the development of either two different IgE molecules for monomeric allergens or single IgE molecules for an oligomeric allergen^8^,^9^. Thus, the presentation of the allergen as an amyloid allows the development of a single IgE molecule and the optimization by enhancing the probability of the cross-linking step.

The phase transition accompanying amyloid formation has also major implications in allergy diagnosis. Clearing amyloids (either by their physical removal or by using additives that prevent their formation) from solutions used for skin prick tests may result in false negatives compared with non-risk-free oral food challenges^8^. This strategy is of particular relevance for cases in which amyloids are preferentially formed after the partial digestion of food allergens^36^,^49^. Therefore, in light of these results, a food allergy diagnosis should be implemented for the consideration of allergen folds displaying insolubility.

## Methods

### Ethics statement

Approval from the Ethics Committee (PI1950) of Hospital Universitario La Paz (Madrid, Spain) was obtained. Parents signed written informed consent, and in the case of children aged 12 or older, assent from the children was also obtained. All methods were performed in accordance with the guidelines of the Hospital Universitario La Paz.

### Patient sera

Sera samples from 10 fish-allergic patients (mean age: 9.8 years, 7 boys) from the Hospital Universitario La Paz with specific IgE antibodies to cod parvalbumin were selected. All patients had a history of symptoms suggestive of immediate hypersensitivity elicited by eating fish, with positive skin prick tests to fish extracts: cod and tuna (1000 IC/mL; Alyostal, Stallergenes); swordfish and salmon (1 mg/mL; Bial Aristegui); hake (1.25 mg/mL; Laboratorios LETI S.L.) and megrim (1 mg/mL; Laboratorios LETI S.L.), as well as specific IgE antibodies to fish, as determined by the CAP-System FEIA^TM^ (ThermoFisher) (Supplementary Table S1).

### Proteins and peptides

rGad m 1 (recombinant Gad m 1) was produced from a pET15b construct containing the synthetic ORF of *Atlantic cod* parvalbumin A51874^36^. The mutants were prepared using Quick-Change protocols and a pair of complementary oligonucleotides (Supplementary Table S2). The proteins were produced in BL21(DE3) cells and purified from the soluble fraction by Ni^2+^-affinity chromatography (GE Healthcare Life Sciences), followed by Q-Sepharose chromatography (GE Healthcare Life Sciences)^36^. The eluted fractions were filtered using 30 kDa-pore size Amicon Ultra-15 (Merck Millipore) and extensively dialyzed against 5 mM Hepes, pH 7.5, containing 0.1 mM CaCl_2_^36^. Before use, the proteins were centrifuged at 16,000 × *g* for 20 min at 4 °C to remove any insoluble material. Protein concentrations were determined using the Bradford protein assay (Bio-Rad) calibrated with BSA (Sigma, A8806). Aβ42 was obtained from GenScript and used as previously described^57^.

### Peptide arrays (SPOT)

Dodecapeptides spanning the whole sequence of Gad m 1, with an overlap of ten residues, were solid-phase synthesized and immobilized (≈10 nmol per spot) on an Amino-PEG500-UC540 sheet at the National Centre for Biotechnology (CNB-CSIC, Madrid). Before use, the membranes were rinsed with ethanol, washed three times with TBS (25 mM Tris-HCl, pH 7.5, containing 137 mM NaCl and 2.7 mM KCl), and incubated in TBS containing 1% BSA (w/v) and 2 mM EDTA for 1 h. The membranes were then probed for 2 h with sera from patients who are allergic to fish (1/10 dilution) and the anti-amyloid fibril OC antibody (AB2286 Merck Millipore, 1/2,000 dilution), prepared in TBST (TBS containing 0.05% Tween-20) supplemented with 0.5% BSA (w/v) or with rGad m 1 chains (2.5 μM) in TBS. After extensive washes with TBST, a 30 min incubation with either horseradish peroxidase-labeled anti-human IgE (Abcam ab99806, 1/2,000 dilution), anti-human IgG4 (Abcam ab99823, 1/4,000 dilution), goat anti-rabbit IgG (Sigma, 1/5,000 dilution) or anti-6X His tag® antibody (Abcam ab18184, dilution 1/1,000) was performed. The signal was developed with the ECL-Western-blotting reagent (Bio-Rad) and detected with a ChemiDoc XRS instrument^36^. When required, the membranes were regenerated by sequential incubation with TBS containing 8 M urea, 1% SDS and 0.5% β-mercaptoethanol for 30 min at 55  °C and three times with acetic acid/ethanol/Milli-Q water (10:50:40). Regions were considered major allergenic epitopes when at least two overlapping peptides were involved. Signals resulting from the binding of secondary antibodies in the absence of primary antibodies were negligible under the conditions tested.

### Circular Dichroism Spectroscopy

Circular dichroism (CD) experiments were performed using a Jasco J-820 spectropolarimeter equipped with a Peltier-controlled thermostatted cell holder. Far UV CD spectra were recorded for a 25 μM protein concentration in 50 mM Tris-HCl, pH 7.5, supplemented with either 1 mM EDTA or 1 mM CaCl_2_. Thermal denaturation experiments were performed to follow the ellipticity changes at 222 nm upon heating from 15 °C to 90 °C at a 1 degree/min heating rate. Both the spectra and thermal unfolding curves were analyzed as previously described^36^,^58^.

### Dynamic Light Scattering

Dynamic light scattering (DLS) measurements were performed using a DynaPro spectroscatter (Wyatt Technology) with a 1.5-mm path length and a 12 μl quartz cuvette. The average of 20–25 acquisitions of buffers and protein solutions (160 μM protein concentrations) were filtered using a 0.2 μm Whatman Anodisc-3 filter. The hydrodynamic radii (R_H_) and mass proportions (%) of the species were derived as previously described^36^,^58^. The experiments were performed in duplicate using two different protein preparations. The theoretical hydrodynamic radius R_H_^T^ for spherical rGad m 1 was calculated as 1.81 nm using 0.73 cm^3^ g^−1^ and 0.35 g of H_2_O (g protein)^−1^ for the particle-specific volume and hydration^36^,^58^.

### Amyloid formation assays

The amyloid propensity and location of the segments in β-parvalbumin chains with adhesive properties was theoretically evaluated using the ZipperDB algorithm, as previously described^36^. rGad m 1 wt and the mutants were prepared at a concentration of 150 μM in 50 mM Tris-HCl, pH 7.5, with 0.1 M NaCl, supplemented with either 5 mM EDTA or 5 mM CaCl_2_. The kinetics of thioflavin T (ThT) binding was monitored by bottom reading the fluorescence intensity in a POLARstar microplate reader (BMG Labtech), as previously described^36^,^57^,^58^. The measurements were performed using 0.18 mL samples containing 10 μM ThT and 450 nm excitation and 480 nm emission filters. The measurement program consisted of 10 flashes, a reading collected every 15 min, 0.5-min of orbital shaking at 100 rpm, and the temperature controller was set to 37 °C. All measurements were collected in duplicate, and the experiments were repeated at least twice using two different protein batches. When required, the fibers were harvested from the reaction mixtures by centrifugation at 100,000xg for 1 h using an OptimaTm^Max^ Beckman ultracentrifuge. The resulting pellet and supernatant fractions were used for the protein and ThT determinations.

### Atomic force microscopy

rGad m 1 wt fibrils were deposited onto freshly cleaved mica surfaces for 10 min, washed with H_2_O, and dried with a stream of N_2_. AFM images were recorded in a MultiMode Veeco microscope and analyzed using WSxM (Nanotec), as previously described^36^,^58^.

### Dot-blot analysis

The immunoreactivity of the rGad m 1 wt and mutant species was assessed by dot-blot analysis using the anti-amyloid fibril OC antibody (AB2286 Merck Millipore, 1/2,000 dilution) and sera from patients who are allergic to fish (1/10 dilution). Briefly, aliquots containing 50–100 ng of protein in the different states were spotted in duplicate on a nitrocellulose membrane. Immunodetection was performed by incubating the membranes with the primary antibodies for 1 h, followed by extensive washes and 30 min incubation with horseradish peroxidase-labeled either mouse monoclonal B3102E8 anti-human IgE (Abcam, diluted 1/2,000) or goat anti-rabbit IgG (Sigma, diluted 1/5,000). The signal was developed using the ECL-Western-blotting reagent (Bio-Rad) and detected with a ChemiDoc XRS instrument^36^.

### ELISA assays

Polystyrene 96-well plates (Costar 3590SA) were coated with 100 μl of rGad m1 chains in 0.2 M carbonate buffer, pH 9.4, by varying the concentrations from 0.1–500 μg/ml for 2 h at 37 °C. The coated wells were blocked with 1% BSA in TBS for 30 min and then incubated with 100 μl of a 1:10 dilution of the patients’ serum in TBST containing 1% BSA for 2 h at room temperature. After washing with TBST, the wells were incubated with peroxidase-labeled anti human IgE (Abcam, diluted 1:2,000) for 1.5 h at room temperature. The plates were washed again and then developed with 100 μl of TMB-turbo ELISA substrate (Thermo Scientific). The reaction was stopped after 30 min with 10 μl of 2N H_2_SO_4_ and the optical density (OD) was measured at 450 nm using a microplate reader (Bio-Rad 3550). The assays were performed in duplicate using blocking buffer as negative control and were statistically analyzed and fitted using Origin software. A parallel analysis using the Bradford protein assay (Bio-Rad) indicated similar extent of adsorption for all proteins used.

### Protease digestion and mass spectrometry

Typically, 50 μl of rGad m 1 fibrils (160 μM) were digested in 10 mM Gly-HCl, pH 1.3, with pepsin (1:50 w:w protease/substrate) at 37 °C for 1 h. The reactions were stopped by the addition of 1 M Tris-HCl, pH 8. After disaggregation with hexafluorisopropanol (HIFP), the hydrolyzed products were analyzed by mass spectrometry using a CHCA matrix and a MALDI-TOF/TOF 4800 (SCIEX, reflector mode) for identification and HPLC (Eksigent Technologies nanoLC Ultra 1D plus, SCIEX) coupled to a Triple TOF 5600 (SCIEX) for fragmentation at the CNB Proteomics Facility (CNB-CSIC, Madrid).

## Additional Information

**How to cite this article**: Sánchez, R. *et al*. The amyloid fold of Gad m 1 epitopes governs IgE binding. *Sci. Rep.* **6**, 32801; doi: 10.1038/srep32801 (2016).

## Supplementary Material

Supplementary Information
