# Supplementary material for: The amyloid fold of Gad m 1 epitopes governs IgE binding
Source: Sci Rep. 2016 Sep 6;6:32801. doi: 10.1038/srep32801 (PMC5011719; doi:10.1038/srep32801)

The amyloid fold of Gad m 1 epitopes governs IgE binding

Rosa Sánchez[1], Javier Martínez[1]*, Ana Castro[2], María Pedrosa[3,4], Santiago Quirce[3,4], Rosa Rodriguez-Perez[4] and María Gasset[1]**

[1]Instituto de Química Física "Rocasolano", Consejo Superior de Investigaciones Científicas, 28006 Madrid, Spain.
[2]Instituto de Química Médica, Consejo Superior de Investigaciones Científicas, 28006 Madrid, Spain.
[3]Departamento de Alergología, Hospital Universitario La Paz, 28046 Madrid, Spain
[4]Instituto de Investigación Hospital Universitario La Paz (IdiPaz), 28046 Madrid, Spain

***Present address:** Biosynthesis and Integrative Science Institute and Department of Chemistry and Biochemistry, Facultade de Ciências, Universidade de Lisboa. 1749-016 Lisboa, Portugal.

****Corresponding author:** María Gasset, maria.gasset@csic.es

Table S1. Clinical data and sera features of the 10 patients allergic to fish.

Patient	Age (years)	Sex[a]	Symptoms after fish ingestion[b]	Offending fish[c]	Other food allergies	Cod SPT (mean diameter, mm)[d]	Total IgE (kU/l)	Cod sIgE (kU/l)
**S1**	12.5	M	OAS	Hake, megrim	-	8	776	12.2
**S2**	9.1	M	U, OAS	Hake, tuna	Seafood, tree nuts	12	1020	13.9
**S3**	9.4	F	U	Megrim	-	0	1843	3.4
**S4**	10.3	F	U, AE, OAS, V	Hake, megrim	Fruits	5	851	8.4
**S5**	8.6	M	U, V	Hake, cod	Egg, fruits, tree nuts	4	1054	3.7
**S6**	8.5	M	U, AE, BS	Hake, megrim	Egg, seafood, fruits, tree nuts	5.5	4277	7.5
**S7**	10.3	M	V	Hake, cod, megrim	Egg, seafood, legumes, tree nuts	6	2223	8.0
**S8**	4.6	M	AE, U	Hake	Egg	15	901	15.3
**S12**	16.4	F	AN	Hake, megrim	Fruits, tree nuts	10.5	516	10.1
**S13**	8.5	M	OAS	Hake, megrim	Egg	14.5	354	27.6

[a] M: male; F: female.
[b] OAS: oral allergy syndrome; U: urticaria; AE: angioedema; V: vomiting; BS: bronchospasm; AN: anaphylaxis.
[c] Cod: *Gadus morhua*; Hake: *Merluccius merluccius*; Megrim: *Lepidorhombus whiffiagonis*; Tuna: *Thunnus albacares*.
[d] SPT: skin prick test.

Table S2. List of primers used in this study for rGad m 1 mutant generation. PV refers to the synthetic ORF of *Atlantic cod* parvalbumin A51874.

Mutation	Template	Primer (forward)
**A**	pET15b-PV	5’-GCGGATGTGAAGACGGCGCTGGAGGCGTG-3´
**C**	pET15b-PV	5’-CGAAAAGCAGCGATGATATTAAGAAGGCGTTTGAAATTATTG-3´
**E**	pET15b-PV	5’-GAGCGATAAGGAAACCAAGGCGTTTCTG-3´
**CE**	pET15b-PVC	5’-GAGCGATAAGGAAACCAAGGCGTTTCTG-3´
**ACE**	pET15b-PVCE	5’-GCGGATGTGAAGACGGCGCTGGAGGCGTG-3´

Figure S1. Sequences and chain location of the 50 overlapping synthetic peptides (12 amino acid lengths, 2 amino acids offset) used for the identification reactive regions in Gad m 1. Peptide numbering (1 to 50) was performed from the N- to the C-terminus of the chain sequence. Helices (A,B,C,D,E and F) forming the three EF hands are represented by blue rectangles, and the Ca^{2+} binding sites depicted as light blue spheres.

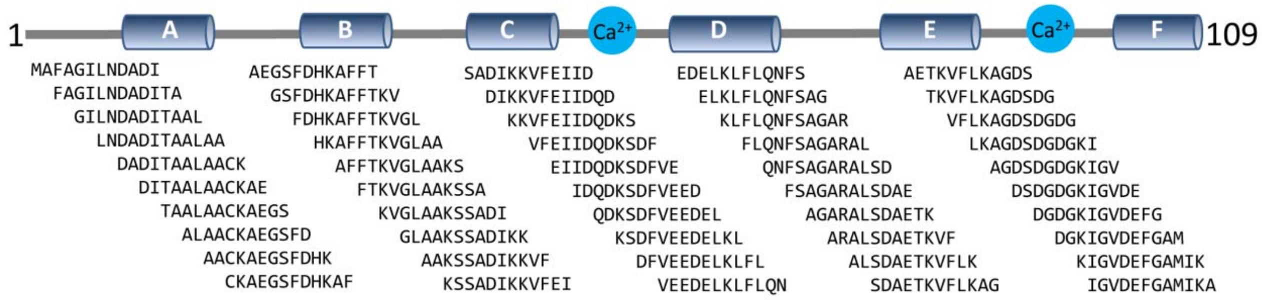

Figure S2. Typical MALDI-TOF/TOF spectra of pepsin-hydrolyzed rGad m 1 amyloids. Mass spectrometry analysis was performed using α-cyano-4-hydroxycinnamic acid (CHCA) matrix. Peak features have been removed for clarity reasons.

.

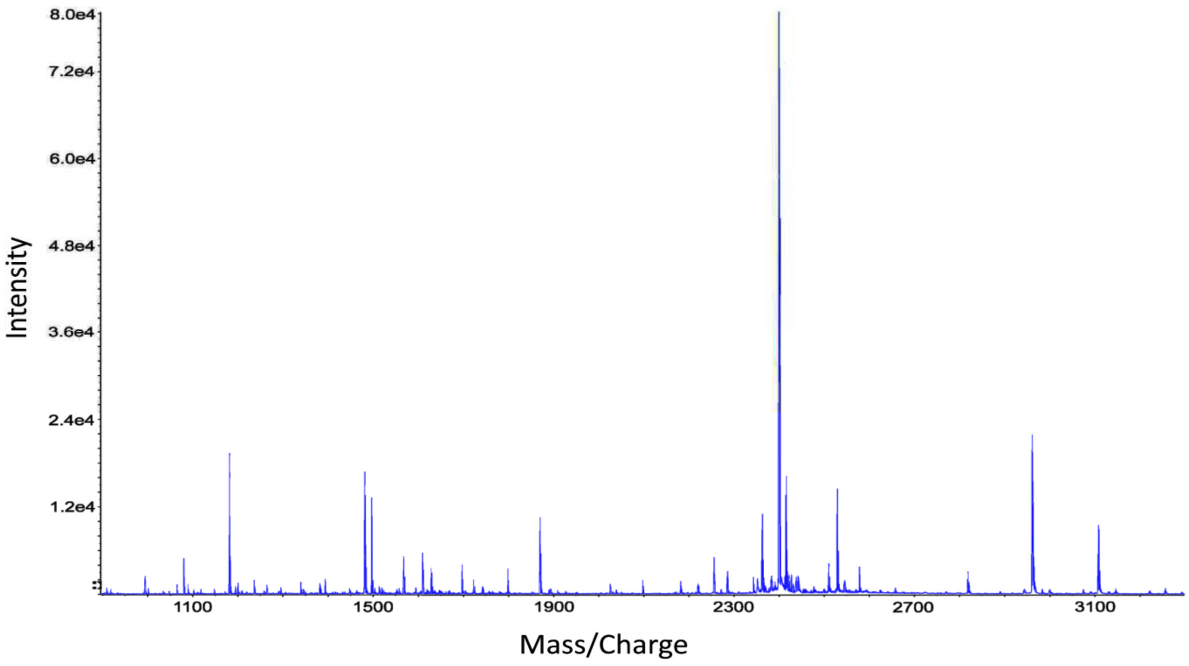

Figure S3. Identification of pepsin-resistant peptides of rGad m 1 amyloids. Fragmentation spectra were obtained using nano-LC coupled to MALDI Triple TOF. Panels (**a**-**e**) depict the distinct sequences that contain undigested pepsin cleavage sites.

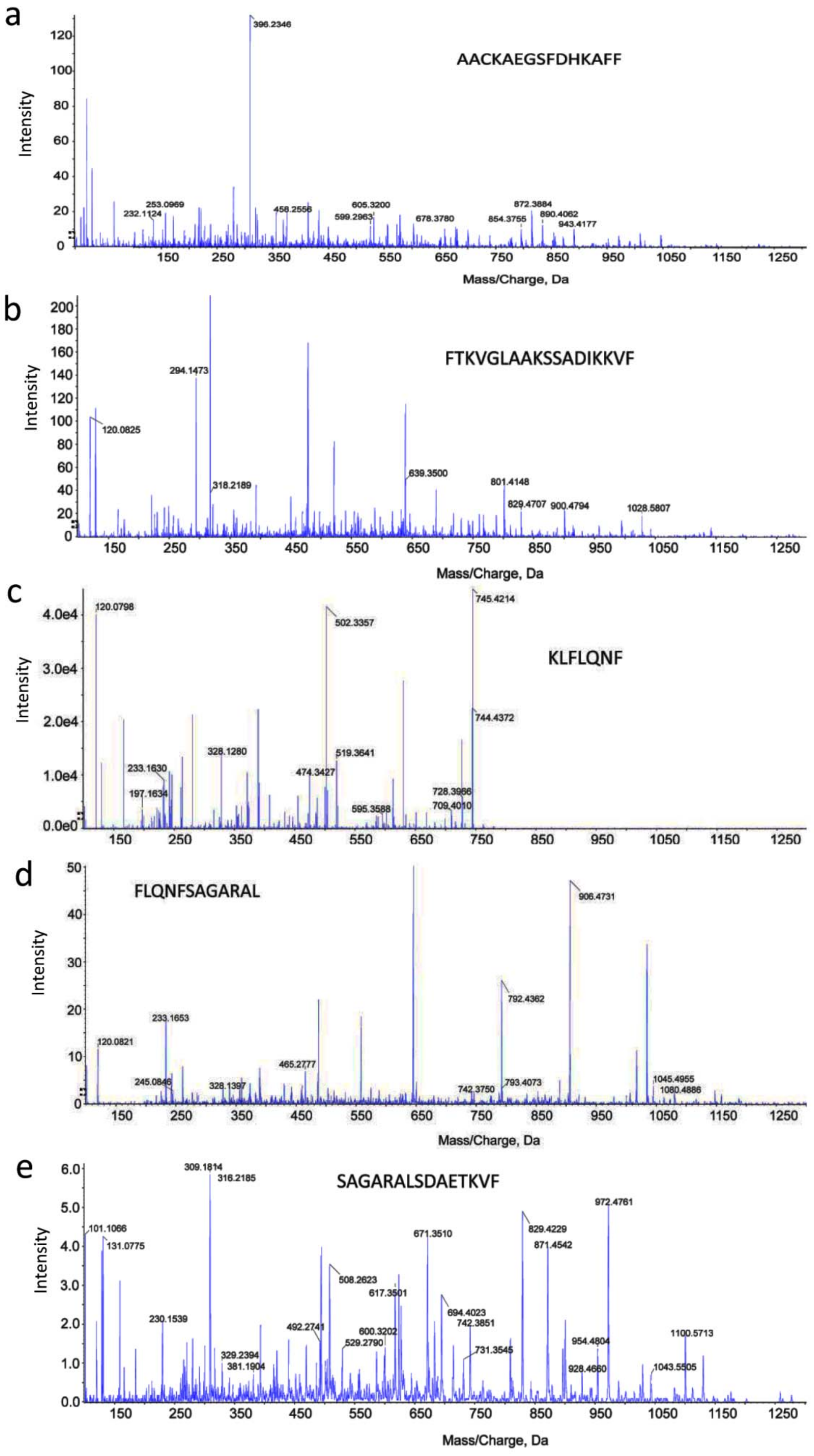

Supplement: Supplementary Information [file srep32801-s1.pdf]
